# Prognosis‐related gene signature is enriched in cancer‐associated fibroblasts in the stem‐like subtype of gastric cancer

**DOI:** 10.1002/ctm2.930

**Published:** 2022-06-26

**Authors:** Ji‐Yong Sung, Jae‐Ho Cheong

**Affiliations:** ^1^ Department of Laboratory Medicine Yonsei University College of Medicine Seoul Korea; ^2^ Department of Surgery Yonsei University College of Medicine Seoul Korea; ^3^ Yonsei Biomedical Research Institute Yonsei University College of Medicine Seoul Korea; ^4^ Department of Biochemistry and Molecular Biology Yonsei University College of Medicine Seoul Korea

Dear Editor

Prognostic markers in gastric cancer^1^ are not only used to clinically classify patients into groups but are also being studied extensively for assessing cancer progression and drug development. Hence, the mechanisms by which genes associated with different prognoses in gastric cancer affect stem‐like molecular subtypes require further investigation. We selected the top 500 genes with significantly different prognostic values for stomach adenocarcinoma in The Cancer Genome Atlas (TCGA) (Figure 1A, Table S1). The genes differed significantly between the high and low expression samples (Figure 1B). Most of these 500 genes were enriched during synapse assembly, trans‐synaptic signalling, and developmental growth (Figure 1C). The frequency of SIG500 detection in each molecular subtype of gastric cancer was determined, and 99% of the genes were found to be present in the stem‐like molecular subtype corresponding to the high group (Figure 1D). The generated Kaplan–Meier plot showed that the group with a high SIG500 score had poor prognosis in three cohorts (Y497, GSE62254 and GSE15459) (Figure 1E). Additionally, we analysed the enriched biological pathways in each cohort in the high SIG500 group. Thus, we observed that in the Yonsei Hospital cohort,^2^ biological pathways related to glycerophospholipid biosynthesis were enriched; in the GSE62254 cohort, pathways related to the M phase were enriched, while in the GSE15459 cohort, pathways related to muscle structure development and the NABA core matrix were enriched (Figure 2A). Thirty‐two master regulators of cancer, blood vessel morphogenesis, NABA core matrisome,^3^ insulin‐like growth factor (IGF) transport, and uptake by IGF binding proteins were enriched in SIG500 genes (Figure 2B). Reportedly, signalling pathways that drive epithelial to mesenchymal transition (EMT) and the IGF1/IGF1 receptor pathway are substantially active in mesenchymal gastric cancer.^4^ Additionally, a high extracellular matrix score is associated with a poor prognosis for gastric cancer,^5^ and proteoglycans and glycosaminoglycans are regulators of cancer stem cell (CSC) function.^6^ In general, the SIG500 genes were enriched in pathways associated with CSCs and the mesenchymal subtype of gastric cancer. We analysed the different immune environments in the high and low SIG500 samples corresponding to four gastric cancer cohorts. Naïve B and inactivated T cells of the innate immunity pathway and immunosuppressive M2 and T regulatory cells were observed in all four cohorts (Figure 2C). High SIG500 samples showed enrichment of EMT, angiogenesis and myogenesis, whereas low SIG500 samples showed enrichment of E2F targets, G2M checkpoint, MYC targets and DNA repair (Figure 2D). We also compared the results of the immune checkpoint inhibitor response,^7^ assuming that high SIG500 is related to the immunosuppressive function associated with adaptive innate immunity, and found that SIG500 was significantly higher in nonresponders (*p* = .007) (Figure 2E). To overcome the limitations associated with bulk samples, we analysed the association between specific cells and SIG500 at the single‐cell level. In total, 12 422 single cells corresponding to eight cell types (cohort 1) and 5927 single cells corresponding to another eight cell types (cohort 2) were analysed as gastric cancer samples. In particular, we observed that the SIG500 score was the highest in fibroblasts, followed by endothelial cells (Figure 3A,B).

**FIGURE 1:**
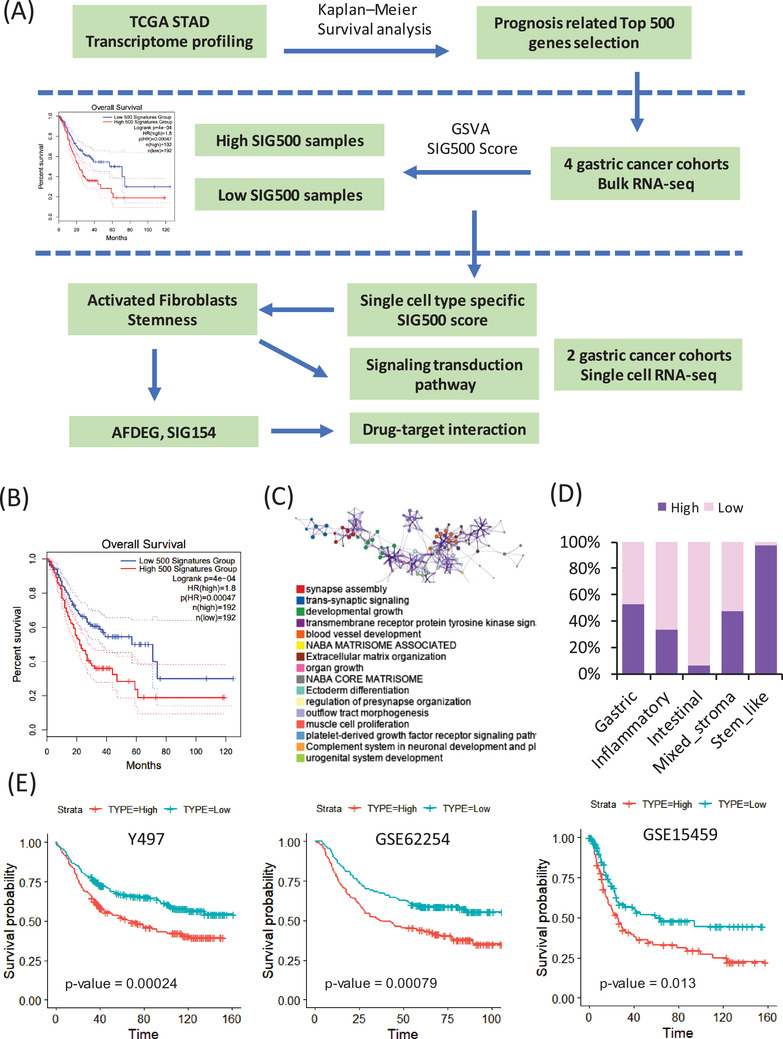
Enrichment of prognosis‐related genes in stem‐like molecular subtypes. (A) Overview of the analysis pipeline. (B) Kaplan–Meier plots showing the overall survival rates for the high and low SIG500 samples in The Cancer Genome Atlas (TCGA) data related to stomach adenocarcinoma. (C) Network of gene ontology analysis of enriched biological pathways related to SIG500. (D) Bar graph showing the SIG500 state across five molecular subtypes in the Y497 cohort. (E) Kaplan–Meier plots showing the overall survival rates for the high and low SIG500 samples. The *p*‐values were determined using the log‐rank test and adjusted using Bonferroni correction. (E) Network of gene ontology analysis for the high SIG500 expression groups in three cohorts (FDR < 0.001). FDR, false discovery rate

**FIGURE 2:**
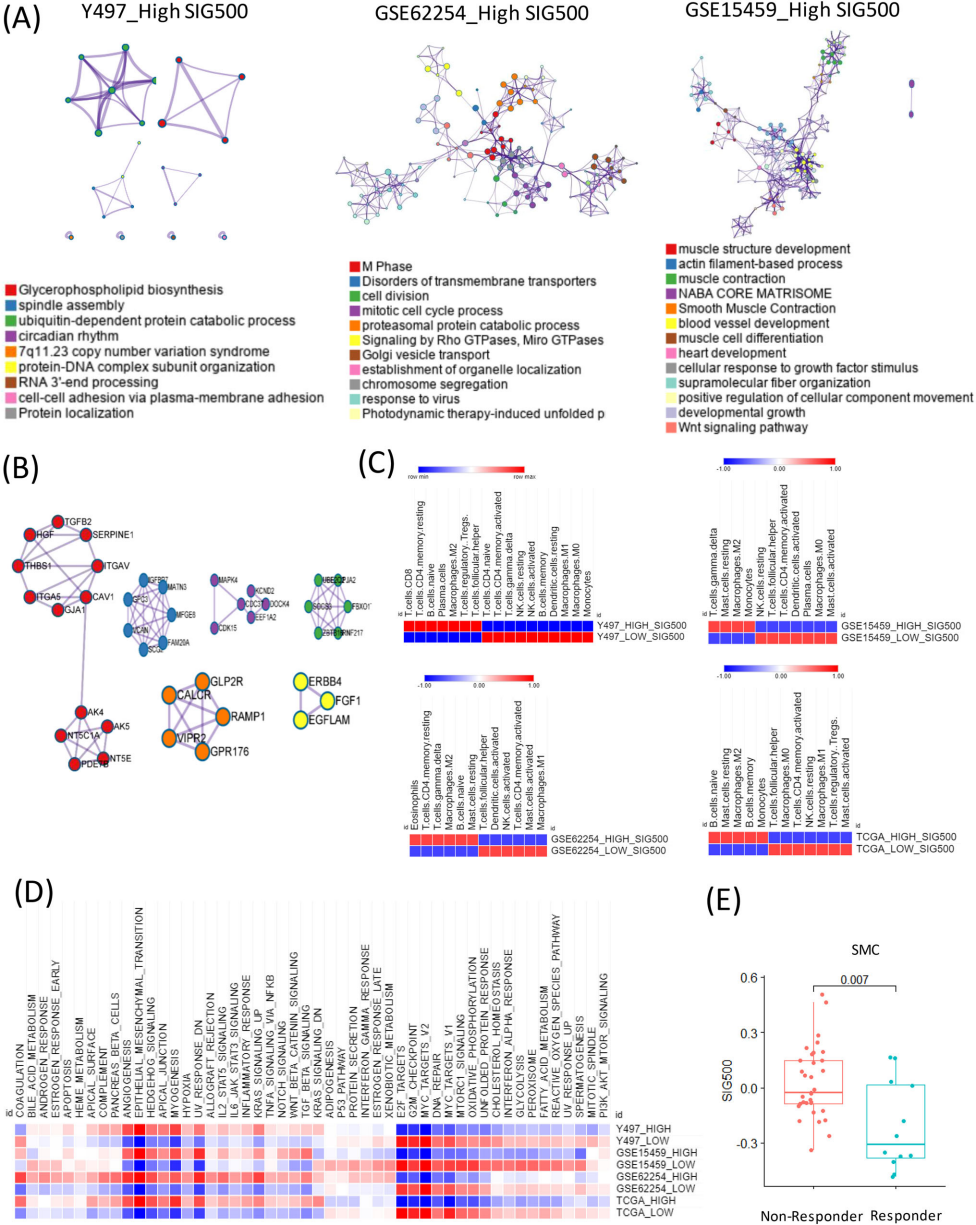
Landscape of cancer hallmarks and biological processes with SIG500. (A) Network of gene ontology analysis for the high SIG500 expression groups in three cohorts (FDR < 0.001). (B) Protein–protein interaction network and MCODE components identified in the SIG500 group. (C) Heatmap showing immune cell types in the high and low SIG500 samples in four cohorts (FDR < 0.001). (D) Heatmap of 50 cancer hallmarks in four cohorts. (E) Boxplot of SIG500 for the immune checkpoint blockade response in Samsung Medical Center Data. FDR, false discovery rate

**FIGURE 3:**
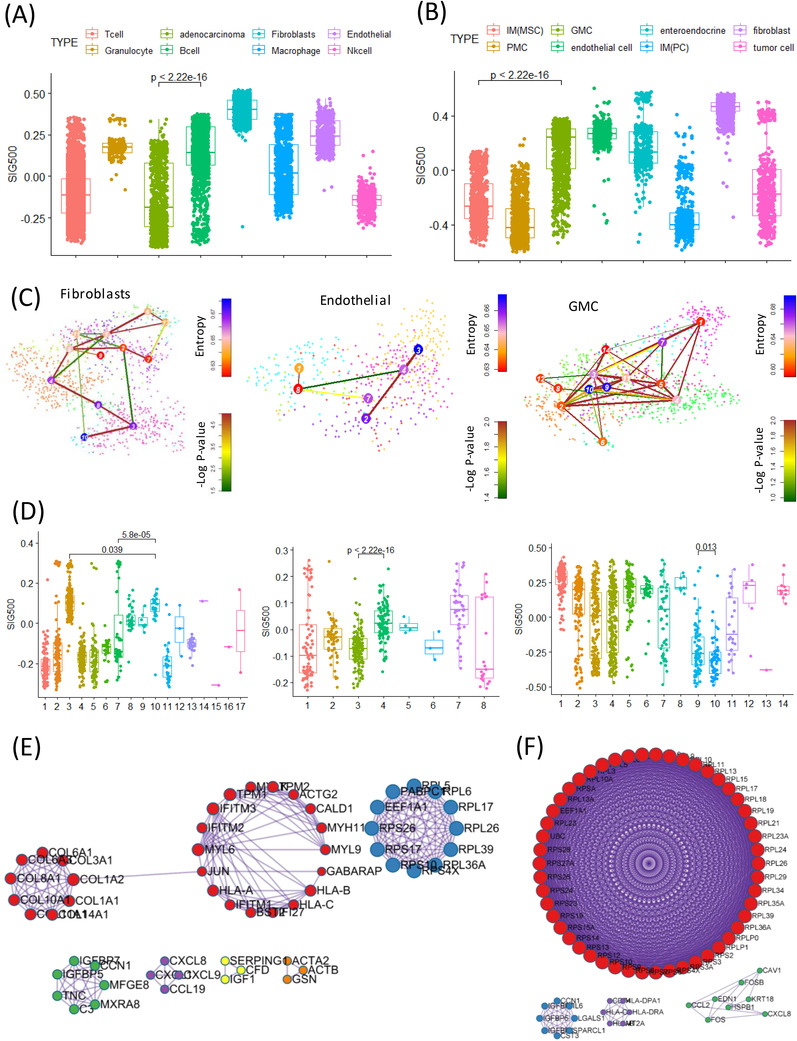
Association between signature 500 genes and specific cell types at the single‐cell level. (A) Boxplot of SIG500 score for eight cell types in cohort 1. (B) Boxplot of SIG500 score for eight cell types in cohort 2. (C) tSNE of fibroblasts, endothelial cells and gland mucous cells (GMCs) for stemness based on StemID. (D) Boxplot of SIG500 score for three single‐cell types (fibroblasts, left; endothelial, middle; GMC, right). (E) Protein–protein interaction network and MCODE components identified for the differentially expressed genes in activated fibroblasts. (F) Protein–protein interaction network and MCODE components identified for the differentially expressed genes in activated endothelial cells. tSNE, t‐distributed stochastic neighbor embedding

We analysed the relationship between cell type stemness and the SIG500 score at the single‐cell level. Thus, we observed that for fibroblasts, the higher the entropy, the lower the SIG500 score; the high fibroblast entropy group (cluster 10) showed significantly higher expression for 154 genes, which were identified as the differentially expressed genes in activated fibroblasts. The high SIG500 score corresponding to nonactivated fibroblasts was indicative of improperly functioning cancer‐associated fibroblasts (CAFs). In this study, CAFs tended to have higher SIG500 scores as stemness increased; however, a group with a lower SIG500 score showing lower stemness was also observed (Figure 3C,D). Gland mucous cells (GMCs) with high stemness had the lowest SIG500 score. Conversely, SIG500 had a high proportion in cells with low stemness. This trend suggested that cells with high GMC stemness are possibly adult stem cells, although we inferred that the expression of SIG500 genes is low before tumourigenesis (Figure 3C,D). In activated fibroblasts with high stemness, the expression of 154 differentially expressed genes (Table S2) was high, and in protein–protein interaction (PPI) analysis using MCODE, NABA core matrix, senescence and autophagy in cancer and smooth muscle contraction were enriched (Figure 3E). In activated endothelial cells, 169 differentially expressed genes were enriched for eukaryotic translational elongation, ribosome cytoplasmic biogenesis and peptide chain elongation (Figure 3F). Furthermore, the pathways enriched in endothelial cells were not related to those associated with SIG500 but to those associated with activated fibroblasts. We performed signal pathway analysis at the single‐cell level. Pathways involved in intercellular communication were predicted using NicheNet.^8^ Thus, LGALS3 was predicted to be the adenocarcinoma ligand, while the ITGB1 fibroblast receptor was predicted to be the fibroblast ligand (Figure 4A,B). Moreover, 154 genes that were significantly and highly expressed in stem‐like activated fibroblasts were identified. SIG154 was also found to be most highly expressed in the stem‐like type of the Y497 bulk sample (Figure 4C). Moreover, these genes were predominantly enriched in the NABA core matrix and during senescence, autophagy in cancer, and smooth muscle contraction, as indicated in the PPI network (Figure 3E). Kaplan–Meier plots showed the overall survival rates corresponding to the high and low SIG154 groups (*p *= .0017) (Figure 4D). Additionally, we analysed 154 gene target–drug interaction networks using CPDB^9^ and predicted several significant candidate target genes, including *PTGS2* (Figure 4E). Drugs such as BI‐2536, GW843682X and S‐trityl‐L‐cysteine were predicted using Genomics of Drug Sensitivity in Cancer (GDSC) (Figure 4F). In the TCGA STAD data set, a high expression level of *PTGS2* was found to be associated with poor prognosis (Figure 4G). Although the drug‐target interaction was low, ligand–receptor analysis indicated that actin and aortic smooth muscle (*ACTA2*) acted as mesenchymal stem‐cell‐ and lineage‐specific markers, indicating that they can be important drug targets. Our results also indicated for the first time that 154 activated fibroblast‐related genes contribute to the establishment of a stem‐like molecular subtype.

**FIGURE 4:**
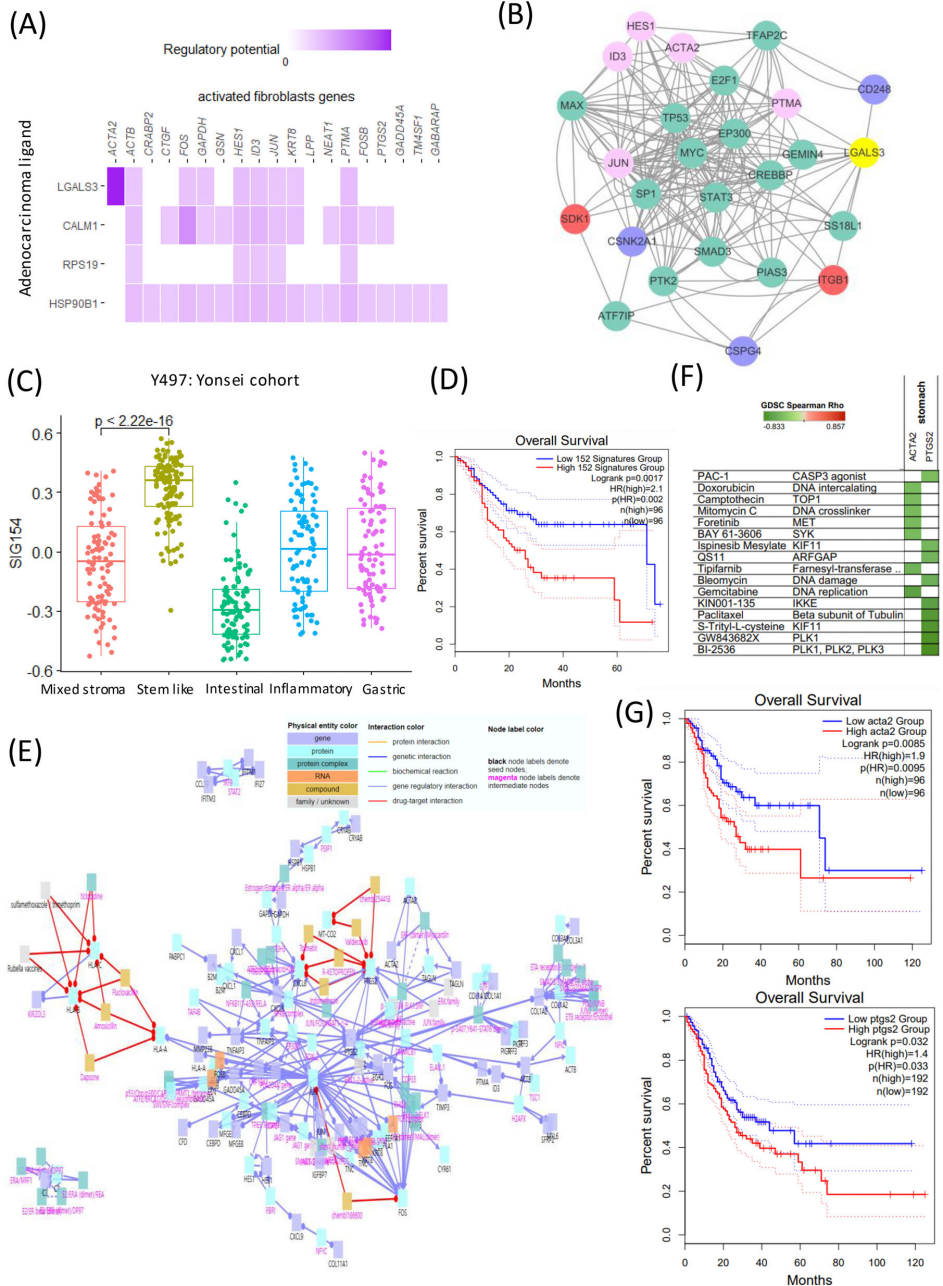
Therapeutic implications of activated fibroblasts in stem‐like type refractory gastric cancer treatment. (A) Heatmap of ligand and SIG500 target genes at the single‐cell level in cohort 1. (B) Network of signal transduction pathways between adenocarcinoma and fibroblasts for activating genes in fibroblasts in single‐cell cohort 1. (C) Differentially expressed genes of activated fibroblasts (SIG152) in five molecular subtypes in Y497. (D) Kaplan–Meier plots showing the overall survival rates for the high and low SIG154 groups (protein–protein interaction [PPI] MCODE genes). (E) Drug–target interaction network for SIG152. (F) Drug and target genes for stomach cancer determined using GDSC. Green colour (negative correlation) shows high drug sensitivity. (G) Kaplan–Meier plots showing the overall survival rates for the high and low *PTGS2* and *ACTA2* expression groups

## CONFLICT OF INTEREST

The authors declare that they have no competing interests.

## Supporting information

Supplementary material

Figure S1. Single‐cell analysis for stemness and SIG500. (A) Stemness for B cells. (B) Transition probability of stemness for B cells. (C) Boxplot of SIG500 for B cells. (D) Boxplot of EBI3 expression for B cells. (E) Boxplot of SIG500 for the transition cluster of B‐cell stemness. (F) Stemness for macrophages. (G) Transition probability of stemness for macrophages. (H) Boxplot of SIG500 for macrophages. (I) Boxplot of CD163 expression for macrophages. (J) Boxplot of SIG500 for the transition cluster of macrophage stemness

Figure S2. Prioritised macrophage ligands and receptors expressed by fibroblasts

Table S1. SIG500 gene list

Table S2. Activated fibroblast gene list

Supplementary material

## References

[1] Kulig P , Nowakowski P , Sierzęga M , et al. Analysis of prognostic factors affecting short‐term and long‐term outcomes of gastric cancer resection. Anticancer Res. 2021;41(7):3523‐3534. 34230148 10.21873/anticanres.15140

[2] Cheong J‐H , Yang H‐K , Kim H , et al. Predictive test for chemotherapy response in resectable gastric cancer: a multi‐cohort, retrospective analysis. Lancet Oncol. 2018;19(5):629‐638. 29567071 10.1016/S1470-2045(18)30108-6

[3] Sung JY , Cheong JH . The matrisome is associated with metabolic reprograming in stem‐like phenotypes of gastric cancer. Cancers. 2022;14(6). 10.3390/cancers14061438 PMC8945874 35326589

[4] Oh SC , Sohn BH , Cheong J‐H , et al. Clinical and genomic landscape of gastric cancer with a mesenchymal phenotype. Nat Commun. 2018;9(1):1777. 29725014 10.1038/s41467-018-04179-8 PMC5934392

[5] Yang Z , Xue F , Li M , et al., Extracellular matrix characterization in gastric cancer helps to predict prognosis and chemotherapy response. Front Oncol. 2021;11:753330. 34646782 10.3389/fonc.2021.753330 PMC8503650

[6] Vitale D , Kumar Katakam S , Greve B , et al. Proteoglycans and glycosaminoglycans as regulators of cancer stem cell function and therapeutic resistance. FEBS J. 2019;286(15):2870‐2882. 31230410 10.1111/febs.14967

[7] Kim ST , Cristescu R , Bass AJ , et al. Comprehensive molecular characterization of clinical responses to PD‐1 inhibition in metastatic gastric cancer. Nat Med. 2018;24(9):1449‐1458. 30013197 10.1038/s41591-018-0101-z

[8] Browaeys R , Saelens W , Saeys Y . NicheNet: modeling intercellular communication by linking ligands to target genes. Nat Methods. 2020;17(2):159‐162. 31819264 10.1038/s41592-019-0667-5

[9] Kamburov A , Stelzl U , Lehrach H , Herwig R . The ConsensusPathDB interaction database: 2013 update. Nucleic Acids Res. 2013;41(Database issue):D793‐D800. 23143270 10.1093/nar/gks1055 PMC3531102

