# Supplementary material for: Prognosis‐related gene signature is enriched in cancer‐associated fibroblasts in the stem‐like subtype of gastric cancer
Source: Clin Transl Med. 2022 Jun 26;12(6):e930. doi: 10.1002/ctm2.930 (PMC9234682; doi:10.1002/ctm2.930)
Supplement: Supplementary file 1 — Supplementary material [file CTM2-12-e930-s005.docx]

**Supplementary Table1.** SIG500 gene list

**Supplementary Table2.** Activated fibroblasts gene list

**Supplementary Figure1.** Single cell analysis for stemness and SIG500 (A) Stemness for B cells (B) Transition probability of stemness for B cells (C) Boxplot of Sig500 for B cells (D) Boxplot of EBI3 expression for B cells (E) Boxplot of SIG500 for transition cluster of B cell stemness (F) Stemness for macrophages (G) Transition probability of stemness for macrophages (H) Boxplot of SIG500 for macrophages (I) Boxplot of CD163 expression for macrophages (J) Boxplot of SIG500 for transition cluster of macrophages stemness

**Supplementary Figure2.** Prioritized macrophage ligands and receptors expressed by fibroblasts
