# Supplementary figures and images for: Prognosis‐related gene signature is enriched in cancer‐associated fibroblasts in the stem‐like subtype of gastric cancer
Source: Clin Transl Med. 2022 Jun 26;12(6):e930. doi: 10.1002/ctm2.930 (PMC9234682; doi:10.1002/ctm2.930)

A.

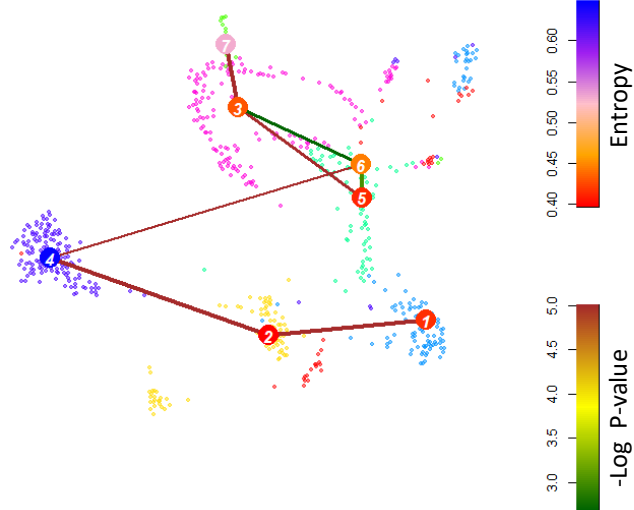

B.

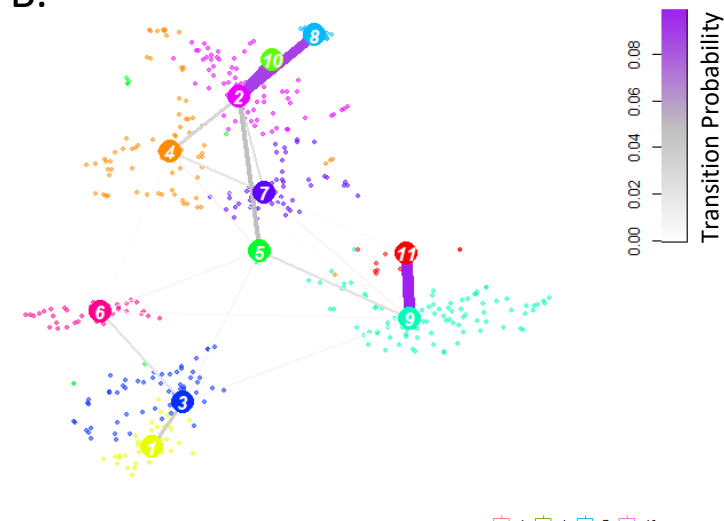

C.

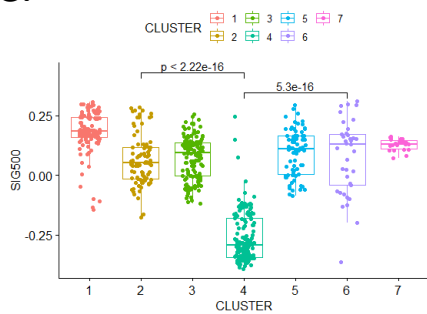

D.

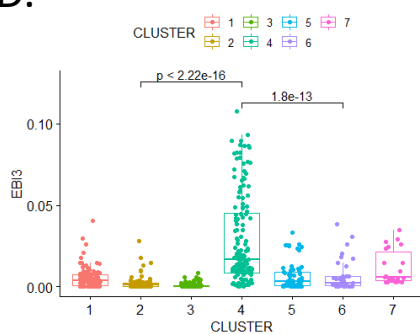

E.

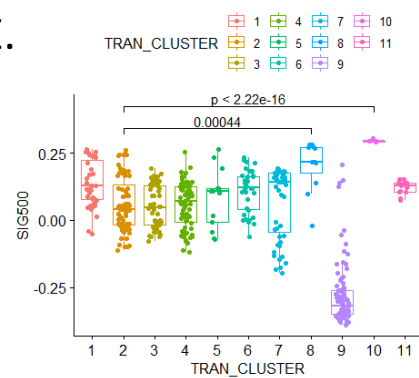

F.

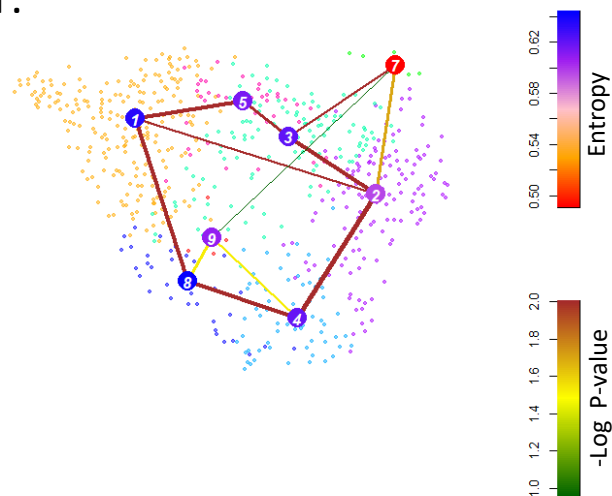

G.

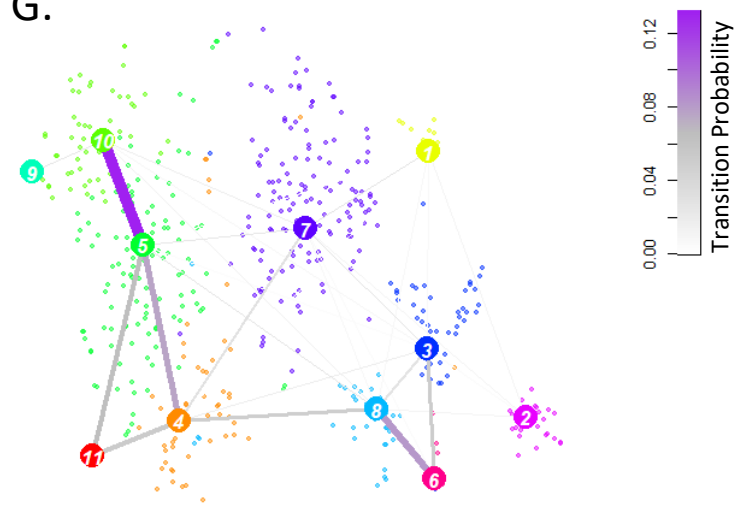

H.

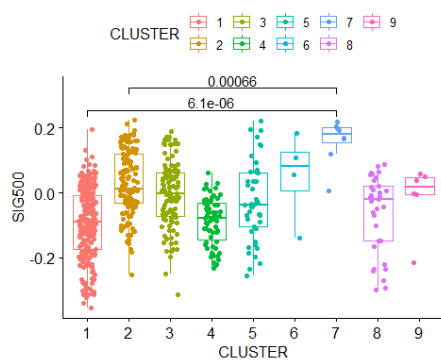

I.

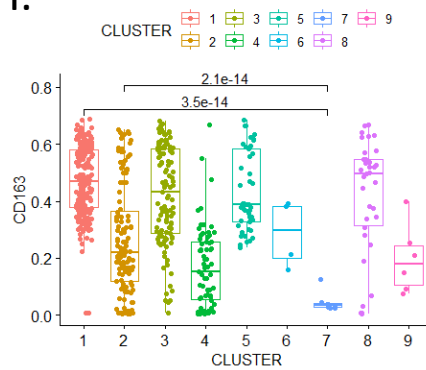

J.

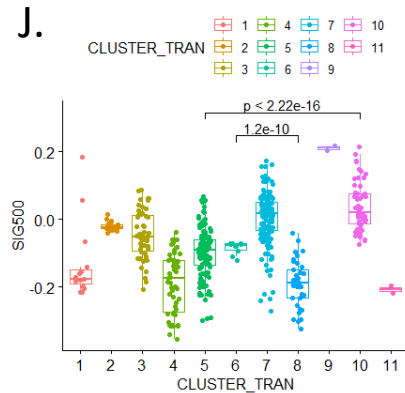

Supplement: Supplementary file 2 — Figure S1. Single‐cell analysis for stemness and SIG500. (A) Stemness for B cells. (B) Transition probability of stemness for B cells. (C) Boxplot of SIG500 for B cells. (D) Boxplot of EBI3 expression for B cells. (E) Boxplot of SIG500 for the transition cluster of B‐cell stemness. (F) Stemness for macrophages. (G) Transition probability of stemness for macrophages. (H) Boxplot of SIG500 for macrophages. (I) Boxplot of CD163 expression for macrophages. (J) Boxplot of SIG500 for the transition cluster of macrophage stemness [file CTM2-12-e930-s002.pdf]
