# Supplementary material for: Prognosis‐related gene signature is enriched in cancer‐associated fibroblasts in the stem‐like subtype of gastric cancer
Source: Clin Transl Med. 2022 Jun 26;12(6):e930. doi: 10.1002/ctm2.930 (PMC9234682; doi:10.1002/ctm2.930)
Supplement: Supplementary file 3 — Figure S2. Prioritised macrophage ligands and receptors expressed by fibroblasts [file CTM2-12-e930-s003.pdf]

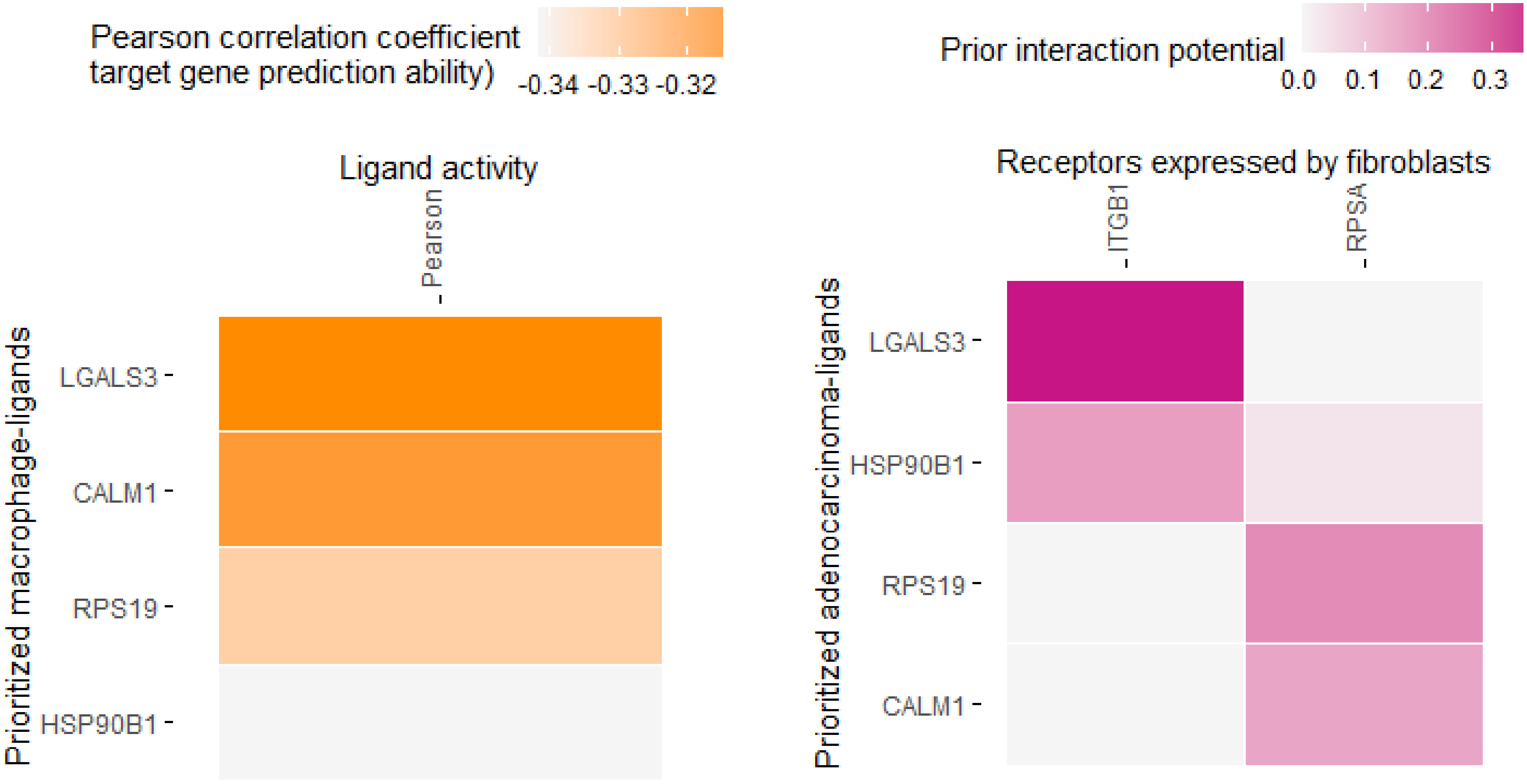

Pearson correlation coefficient
target gene prediction ability)
-0.34 -0.33 -0.32
Ligand activity
Pearson
Prioritized macrophage-ligands
LGALS3
CALM1
RPS19
HSP90B1
Prior interaction potential
0.0 0.1 0.2 0.3
Receptors expressed by fibroblasts
ITGB1
RPSA
Prioritized adenocarcinoma-ligands
LGALS3
HSP90B1
RPS19
CALM1
