# Supplementary material for: Prognosis‐related gene signature is enriched in cancer‐associated fibroblasts in the stem‐like subtype of gastric cancer
Source: Clin Transl Med. 2022 Jun 26;12(6):e930. doi: 10.1002/ctm2.930 (PMC9234682; doi:10.1002/ctm2.930)
Supplement: Supplementary file 4 — Table S1. SIG500 gene list [file CTM2-12-e930-s004.pdf]

ID	Gene ID	P-Value (Survival os)
GFAP	ENSG0000	2.12E-05
RP11-497E19.1	ENSG0000	2.33E-05
ASPA	ENSG0000	2.69E-05
SERPINE1	ENSG0000	3.41E-05
ZNF883	ENSG0000	3.59E-05
AOC4P	ENSG0000	3.86E-05
CBLN4	ENSG0000	3.97E-05
NT5E	ENSG0000	4.74E-05
AC002480.3	ENSG0000	6.37E-05
MEI4	ENSG0000	6.39E-05
CTD-2054N24.2	ENSG0000	6.93E-05
RP11-1069G10.2	ENSG0000	9.09E-05
EMX2OS	ENSG0000	9.24E-05
ZNF192P1	ENSG0000	9.45E-05
RAI14	ENSG0000	9.67E-05
TRHDE-AS1	ENSG0000	1.07E-04
GUCY1A2	ENSG0000	1.10E-04
PCDHB17P	ENSG0000	1.12E-04
PLCXD3	ENSG0000	1.33E-04
RP11-102K13.5	ENSG0000	1.36E-04
RP11-322E11.5	ENSG0000	1.40E-04
SORCS3	ENSG0000	1.75E-04
AKR1B1	ENSG0000	1.80E-04
CDR1	ENSG0000	1.90E-04
NRP1	ENSG0000	2.03E-04
PJA2	ENSG0000	2.17E-04
SLITRK2	ENSG0000	2.32E-04
INHBB	ENSG0000	2.34E-04
LIFR-AS1	ENSG0000	2.39E-04
CPT1C	ENSG0000	2.65E-04
GOLGA8IP	ENSG0000	2.92E-04
SLC52A3	ENSG0000	3.09E-04
CPNE8	ENSG0000	3.12E-04
PYGO1	ENSG0000	3.13E-04
PKNOX2	ENSG0000	3.29E-04
TFPI2	ENSG0000	3.39E-04
HTR1F	ENSG0000	3.43E-04
LINC00315	ENSG0000	3.53E-04
WDR49	ENSG0000	3.88E-04
SVEP1	ENSG0000	3.92E-04
ERBB4	ENSG0000	3.93E-04
DYNC1I1	ENSG0000	3.98E-04

ITIH3	ENSG0000	4.19E-04
ACOT1	ENSG0000	4.27E-04
MAGED4B	ENSG0000	4.47E-04
FGF1	ENSG0000	4.64E-04
COLEC12	ENSG0000	4.65E-04
CCDC181	ENSG0000	4.70E-04
DGKQ	ENSG0000	4.72E-04
EIF3EP1	ENSG0000	4.84E-04
ATP8A2	ENSG0000	4.86E-04
PPM1E	ENSG0000	4.86E-04
CCDC178	ENSG0000	4.92E-04
AK5	ENSG0000	4.97E-04
PTPRQ	ENSG0000	4.98E-04
MUM1L1	ENSG0000	5.03E-04
BLMH	ENSG0000	5.04E-04
ANKRD6	ENSG0000	5.54E-04
PRTG	ENSG0000	5.68E-04
PCDHB6	ENSG0000	5.94E-04
BASP1	ENSG0000	5.95E-04
ASTN1	ENSG0000	6.06E-04
RP11-469N6.1	ENSG0000	6.24E-04
C16orf47	ENSG0000	6.28E-04
VASH2	ENSG0000	6.30E-04
CTB-31O20.8	ENSG0000	6.31E-04
AC007228.11	ENSG0000	6.33E-04
CDC37	ENSG0000	6.42E-04
GPC3	ENSG0000	6.44E-04
P4HA3	ENSG0000	6.45E-04
CASC10	ENSG0000	6.51E-04
TCEAL7	ENSG0000	6.90E-04
PDK4	ENSG0000	7.06E-04
SGCE	ENSG0000	7.26E-04
MCC	ENSG0000	7.32E-04
MATN3	ENSG0000	7.44E-04
RP11-316O14.1	ENSG0000	7.48E-04
ABCB5	ENSG0000	7.49E-04
ZNF662	ENSG0000	7.51E-04
COL4A5	ENSG0000	7.66E-04
LRFN5	ENSG0000	7.73E-04
RP11-462L8.1	ENSG0000	7.89E-04
IQCA1	ENSG0000	7.95E-04
DPP3	ENSG0000	7.96E-04
RP11-310P5.1	ENSG0000	8.09E-04

DSCAM	ENSG0000	8.13E-04
NPY1R	ENSG0000	8.14E-04
EGFLAM	ENSG0000	8.18E-04
FABP4	ENSG0000	8.20E-04
PCA3	ENSG0000	8.27E-04
ANKRD53	ENSG0000	8.36E-04
ADAMTS18	ENSG0000	8.47E-04
STK32A	ENSG0000	8.87E-04
CSRNP1	ENSG0000	9.08E-04
ABCA8	ENSG0000	9.09E-04
CLRN3	ENSG0000	9.16E-04
NOVA1	ENSG0000	9.38E-04
AC005754.8	ENSG0000	9.43E-04
LINC01436	ENSG0000	9.50E-04
COMMD10	ENSG0000	9.50E-04
PCDHB5	ENSG0000	9.71E-04
PRR15L	ENSG0000	9.75E-04
ARMCX1	ENSG0000	9.77E-04
PCOLCE2	ENSG0000	9.82E-04
NBAT1	ENSG0000	9.87E-04
ABCA4	ENSG0000	1.01E-03
SYN2	ENSG0000	1.01E-03
NAP1L2	ENSG0000	1.04E-03
SYPL2	ENSG0000	1.04E-03
RP11-566K19.6	ENSG0000	1.04E-03
LINC01606	ENSG0000	1.10E-03
SPX	ENSG0000	1.10E-03
AC092667.2	ENSG0000	1.11E-03
CREB5	ENSG0000	1.11E-03
PGM5P3-AS1	ENSG0000	1.13E-03
C1GALT1C1L	ENSG0000	1.14E-03
KCNT2	ENSG0000	1.14E-03
EMX2	ENSG0000	1.15E-03
GPNMB	ENSG0000	1.15E-03
CTD-2525P14.5	ENSG0000	1.17E-03
RP11-295P9.8	ENSG0000	1.19E-03
AC005682.6	ENSG0000	1.19E-03
CD300LG	ENSG0000	1.20E-03
ANXA8	ENSG0000	1.21E-03
FREM1	ENSG0000	1.21E-03
TCEAL5	ENSG0000	1.27E-03
APOD	ENSG0000	1.30E-03
TAL2	ENSG0000	1.32E-03

NLGN4X	ENSG0000	1.32E-03
GULP1	ENSG0000	1.33E-03
TRPC6	ENSG0000	1.34E-03
HOXD1	ENSG0000	1.35E-03
RP11-731F5.1	ENSG0000	1.42E-03
CRTAC1	ENSG0000	1.44E-03
PCDH9	ENSG0000	1.45E-03
PCDHB4	ENSG0000	1.47E-03
CDH6	ENSG0000	1.49E-03
PZP	ENSG0000	1.50E-03
VCAN	ENSG0000	1.50E-03
RERG	ENSG0000	1.54E-03
NALCN	ENSG0000	1.54E-03
STEAP4	ENSG0000	1.54E-03
CNRIP1	ENSG0000	1.54E-03
POU6F2	ENSG0000	1.56E-03
RP11-60A14.1	ENSG0000	1.57E-03
LRCOL1	ENSG0000	1.58E-03
RP3-425C14.4	ENSG0000	1.58E-03
KCND2	ENSG0000	1.59E-03
SYT14	ENSG0000	1.59E-03
AC026904.1	ENSG0000	1.60E-03
RARB	ENSG0000	1.61E-03
CYP27C1	ENSG0000	1.62E-03
RP11-456K23.1	ENSG0000	1.63E-03
CALCR	ENSG0000	1.65E-03
DNM1P46	ENSG0000	1.65E-03
SLC35F1	ENSG0000	1.70E-03
C1orf95	ENSG0000	1.71E-03
LINC01537	ENSG0000	1.73E-03
CADM2	ENSG0000	1.73E-03
AP000892.6	ENSG0000	1.74E-03
ITPRIPL1	ENSG0000	1.74E-03
HOXA10-AS	ENSG0000	1.77E-03
ACSM5	ENSG0000	1.77E-03
RBMS1	ENSG0000	1.81E-03
NOXO1	ENSG0000	1.81E-03
CPA6	ENSG0000	1.83E-03
SLC2A3	ENSG0000	1.85E-03
RAB34	ENSG0000	1.85E-03
FAM20A	ENSG0000	1.86E-03
RASSF8	ENSG0000	1.87E-03
RP11-567J20.2	ENSG0000	1.87E-03

ZFHX4	ENSG0000	1.88E-03
MPP4	ENSG0000	1.89E-03
EIF2AK4	ENSG0000	1.89E-03
ZNF22	ENSG0000	1.92E-03
PDLIM1P4	ENSG0000	1.92E-03
MFAP3L	ENSG0000	1.92E-03
SRMS	ENSG0000	1.94E-03
ZNF415P1	ENSG0000	1.95E-03
TSPAN5	ENSG0000	1.96E-03
ASF1B	ENSG0000	1.96E-03
AC091878.1	ENSG0000	1.97E-03
RP11-211N11.5	ENSG0000	1.98E-03
NAT8L	ENSG0000	1.98E-03
NECAB1	ENSG0000	1.99E-03
ZNF331	ENSG0000	2.00E-03
FOLR3	ENSG0000	2.01E-03
EHD3	ENSG0000	2.01E-03
PTCHD4	ENSG0000	2.02E-03
BOLA3-AS1	ENSG0000	2.03E-03
SEC23A	ENSG0000	2.04E-03
IL1RAPL1	ENSG0000	2.08E-03
BEX4	ENSG0000	2.09E-03
AKAP12	ENSG0000	2.09E-03
NRG3	ENSG0000	2.10E-03
LINC00961	ENSG0000	2.11E-03
CLCN3P1	ENSG0000	2.11E-03
CEP83-AS1	ENSG0000	2.11E-03
CHAF1A	ENSG0000	2.11E-03
SCHIP1	ENSG0000	2.11E-03
OSBPL1A	ENSG0000	2.14E-03
CHST14	ENSG0000	2.14E-03
SLC16A7	ENSG0000	2.17E-03
LINC00989	ENSG0000	2.28E-03
ACOT2	ENSG0000	2.30E-03
RP11-620J15.3	ENSG0000	2.31E-03
EPDR1	ENSG0000	2.32E-03
RP11-624L4.1	ENSG0000	2.32E-03
PRL	ENSG0000	2.36E-03
NTN1	ENSG0000	2.37E-03
TCF7L1	ENSG0000	2.38E-03
ITGAV	ENSG0000	2.41E-03
RP11-59D5__B.2	ENSG0000	2.41E-03
ZBTB10	ENSG0000	2.41E-03

GALNT13	ENSG0000	2.43E-03
CDK15	ENSG0000	2.44E-03
MIR99AHG	ENSG0000	2.46E-03
RP11-81K2.2	ENSG0000	2.47E-03
RP11-64C12.8	ENSG0000	2.49E-03
LINC00648	ENSG0000	2.51E-03
LINC00968	ENSG0000	2.52E-03
GLP2R	ENSG0000	2.52E-03
WHAMMP3	ENSG0000	2.53E-03
LGR6	ENSG0000	2.54E-03
NTAN1	ENSG0000	2.56E-03
FAM153B	ENSG0000	2.58E-03
PDE7B	ENSG0000	2.60E-03
ITGA5	ENSG0000	2.61E-03
FAM133A	ENSG0000	2.61E-03
STC1	ENSG0000	2.63E-03
PHF24	ENSG0000	2.63E-03
MSC-AS1	ENSG0000	2.68E-03
RP11-61I13.3	ENSG0000	2.68E-03
NPAS3	ENSG0000	2.68E-03
ADRA1D	ENSG0000	2.68E-03
HAVCR1	ENSG0000	2.68E-03
AC011239.2	ENSG0000	2.70E-03
ADGRL4	ENSG0000	2.74E-03
GOLGA8T	ENSG0000	2.75E-03
ZNF208	ENSG0000	2.76E-03
GPX3	ENSG0000	2.78E-03
LAMA4	ENSG0000	2.79E-03
CCDC23	ENSG0000	2.82E-03
AC020571.3	ENSG0000	2.84E-03
RP11-557L19.1	ENSG0000	2.84E-03
CACNB4	ENSG0000	2.84E-03
KIAA1755	ENSG0000	2.87E-03
RP11-513O13.1	ENSG0000	2.89E-03
ATP2B3	ENSG0000	2.89E-03
LOX	ENSG0000	2.89E-03
ST8SIA6	ENSG0000	2.90E-03
RP11-180I4.4	ENSG0000	2.93E-03
RP11-876N24.5	ENSG0000	2.94E-03
TMEM45A	ENSG0000	2.95E-03
HIF3A	ENSG0000	2.97E-03
TUBA3D	ENSG0000	2.98E-03
CCDC42B	ENSG0000	2.99E-03

ELAVL3	ENSG0000	3.01E-03
SHC4	ENSG0000	3.02E-03
ZNF257	ENSG0000	3.03E-03
MAGED4	ENSG0000	3.03E-03
RASSF8-AS1	ENSG0000	3.06E-03
CELF4	ENSG0000	3.07E-03
AFF3	ENSG0000	3.09E-03
GRTP1	ENSG0000	3.10E-03
BCHE	ENSG0000	3.14E-03
TPST1	ENSG0000	3.15E-03
TIGD6	ENSG0000	3.16E-03
WNK3	ENSG0000	3.18E-03
C6orf48	ENSG0000	3.18E-03
CPNE6	ENSG0000	3.25E-03
TET1	ENSG0000	3.25E-03
MUSK	ENSG0000	3.26E-03
PER1	ENSG0000	3.27E-03
OGN	ENSG0000	3.29E-03
FBXO17	ENSG0000	3.30E-03
RP11-401O9.4	ENSG0000	3.30E-03
AP000476.1	ENSG0000	3.32E-03
LINC00310	ENSG0000	3.33E-03
GFRA2	ENSG0000	3.35E-03
NDUFAF3	ENSG0000	3.35E-03
CTD-2536I1.2	ENSG0000	3.39E-03
MOSPD1	ENSG0000	3.45E-03
PLAT	ENSG0000	3.46E-03
LINC00619	ENSG0000	3.46E-03
C19orf26	ENSG0000	3.49E-03
IGFBP7-AS1	ENSG0000	3.52E-03
ZNF300	ENSG0000	3.54E-03
RP11-553A21.3	ENSG0000	3.56E-03
MDP1	ENSG0000	3.58E-03
NKAIN2	ENSG0000	3.59E-03
ARAP1-AS2	ENSG0000	3.59E-03
MOGAT1	ENSG0000	3.60E-03
AL592528.1	ENSG0000	3.64E-03
EFNA3	ENSG0000	3.65E-03
GPR173	ENSG0000	3.68E-03
GABARAPL2	ENSG0000	3.70E-03
G0S2	ENSG0000	3.70E-03
SV2B	ENSG0000	3.73E-03
RGS4	ENSG0000	3.75E-03

KCNIP1	ENSG0000	3.77E-03
RAMP1	ENSG0000	3.78E-03
THRB	ENSG0000	3.84E-03
AP001626.2	ENSG0000	3.86E-03
AFAP1L1	ENSG0000	3.86E-03
MAGI2-AS3	ENSG0000	3.86E-03
AC016995.3	ENSG0000	3.88E-03
LRAT	ENSG0000	3.88E-03
RP11-681H18.2	ENSG0000	3.90E-03
RP13-514E23.1	ENSG0000	3.93E-03
PCDHB16	ENSG0000	3.95E-03
DOCK4	ENSG0000	3.97E-03
CDO1	ENSG0000	3.99E-03
RP6-109B7.5	ENSG0000	3.99E-03
KIAA1324L	ENSG0000	3.99E-03
USP51	ENSG0000	3.99E-03
CTD-2008L17.2	ENSG0000	4.00E-03
AC004947.2	ENSG0000	4.03E-03
DZIP1	ENSG0000	4.05E-03
TCN2	ENSG0000	4.06E-03
RP11-713P17.3	ENSG0000	4.08E-03
PTPN6	ENSG0000	4.11E-03
IGFBP7	ENSG0000	4.11E-03
AC114730.2	ENSG0000	4.12E-03
RP11-542B15.1	ENSG0000	4.12E-03
CCNA1	ENSG0000	4.12E-03
GPR156	ENSG0000	4.12E-03
RP11-632L2.2	ENSG0000	4.15E-03
MFGE8	ENSG0000	4.18E-03
IGHE	ENSG0000	4.20E-03
RP11-108M12.3	ENSG0000	4.22E-03
VIPAS39	ENSG0000	4.23E-03
HGF	ENSG0000	4.25E-03
SPIRE1	ENSG0000	4.25E-03
ZNF229	ENSG0000	4.26E-03
ZNF423	ENSG0000	4.27E-03
RP11-274H2.3	ENSG0000	4.29E-03
CERS5	ENSG0000	4.30E-03
NACAD	ENSG0000	4.31E-03
BICC1	ENSG0000	4.33E-03
AP4S1	ENSG0000	4.33E-03
GTF2H2C	ENSG0000	4.34E-03
PCDHB3	ENSG0000	4.35E-03

EYA1	ENSG0000	4.38E-03
RP11-600F24.1	ENSG0000	4.38E-03
THBS1	ENSG0000	4.39E-03
QKI	ENSG0000	4.43E-03
PHKG1	ENSG0000	4.45E-03
CHRFAM7A	ENSG0000	4.48E-03
MTTP	ENSG0000	4.50E-03
CD109	ENSG0000	4.52E-03
PRSS3	ENSG0000	4.53E-03
DPT	ENSG0000	4.56E-03
EDNRB	ENSG0000	4.58E-03
KLHDC2	ENSG0000	4.58E-03
HAGLR	ENSG0000	4.60E-03
MSRB3	ENSG0000	4.61E-03
RP11-398K22.12	ENSG0000	4.62E-03
ARMCX2	ENSG0000	4.62E-03
NAP1L6	ENSG0000	4.63E-03
CSMD2	ENSG0000	4.64E-03
DIRC1	ENSG0000	4.66E-03
TREML4	ENSG0000	4.69E-03
ACVR1	ENSG0000	4.69E-03
APBB1	ENSG0000	4.71E-03
ANO4	ENSG0000	4.72E-03
COL24A1	ENSG0000	4.72E-03
C1QTNF4	ENSG0000	4.73E-03
CAND2	ENSG0000	4.73E-03
EEF1A2	ENSG0000	4.74E-03
AK4	ENSG0000	4.75E-03
WDR17	ENSG0000	4.76E-03
LINC00475	ENSG0000	4.78E-03
AC009948.5	ENSG0000	4.79E-03
RP11-155D18.13	ENSG0000	4.79E-03
CLIP3	ENSG0000	4.80E-03
PCDHB13	ENSG0000	4.80E-03
AC000403.4	ENSG0000	4.82E-03
RP3-388E23.2	ENSG0000	4.82E-03
NTMT1	ENSG0000	4.85E-03
ADRA1B	ENSG0000	4.85E-03
RAB19	ENSG0000	4.85E-03
CRB1	ENSG0000	4.85E-03
ZNF474	ENSG0000	4.87E-03
GPR176	ENSG0000	4.87E-03
PSD4	ENSG0000	4.87E-03

LGALS12	ENSG0000	4.88E-03
RPL21P40	ENSG0000	4.89E-03
MYL3	ENSG0000	4.92E-03
NPTX1	ENSG0000	4.95E-03
LINC01140	ENSG0000	4.98E-03
NUDT10	ENSG0000	5.01E-03
POT1-AS1	ENSG0000	5.01E-03
AC002456.2	ENSG0000	5.05E-03
PCCA	ENSG0000	5.07E-03
UBE2QL1	ENSG0000	5.08E-03
C6orf120	ENSG0000	5.08E-03
GLT8D1	ENSG0000	5.10E-03
DIMT1	ENSG0000	5.10E-03
CHRD	ENSG0000	5.10E-03
ZFPM2	ENSG0000	5.13E-03
TGFB2	ENSG0000	5.14E-03
C5	ENSG0000	5.16E-03
DMRTC1B	ENSG0000	5.16E-03
CAV1	ENSG0000	5.17E-03
TNFAIP8L3	ENSG0000	5.19E-03
HSPD1P11	ENSG0000	5.19E-03
EBF2	ENSG0000	5.21E-03
GJA1	ENSG0000	5.21E-03
C1QTNF2	ENSG0000	5.22E-03
PCDHA1	ENSG0000	5.22E-03
CTB-134H23.3	ENSG0000	5.23E-03
FLJ16779	ENSG0000	5.23E-03
LINC00922	ENSG0000	5.24E-03
ARGLU1	ENSG0000	5.24E-03
MASP1	ENSG0000	5.25E-03
SOCS2	ENSG0000	5.29E-03
SOX7	ENSG0000	5.31E-03
CITED2	ENSG0000	5.31E-03
NREP	ENSG0000	5.32E-03
SLITRK4	ENSG0000	5.34E-03
RNF217	ENSG0000	5.34E-03
WASF1	ENSG0000	5.36E-03
SYT6	ENSG0000	5.40E-03
MMP19	ENSG0000	5.41E-03
PTPRD	ENSG0000	5.44E-03
RP11-311D14.1	ENSG0000	5.46E-03
ILDR1	ENSG0000	5.46E-03
REEP4	ENSG0000	5.49E-03

RP4-545K15.5	ENSG0000	5.49E-03
ZRANB2-AS2	ENSG0000	5.51E-03
IL34	ENSG0000	5.52E-03
WBSCR17	ENSG0000	5.52E-03
SV2A	ENSG0000	5.52E-03
KCNK2	ENSG0000	5.58E-03
SIAH3	ENSG0000	5.60E-03
RP11-114H24.2	ENSG0000	5.61E-03
FAM181B	ENSG0000	5.62E-03
PPP1R14A	ENSG0000	5.64E-03
NAMA	ENSG0000	5.66E-03
IL1R1	ENSG0000	5.69E-03
CCNDBP1	ENSG0000	5.69E-03
RAB9B	ENSG0000	5.70E-03
PDGFD	ENSG0000	5.71E-03
ZC3H12C	ENSG0000	5.71E-03
APOC3	ENSG0000	5.71E-03
RP11-284F21.7	ENSG0000	5.72E-03
UBE2Q2	ENSG0000	5.75E-03
STARD9	ENSG0000	5.75E-03
NT5C1A	ENSG0000	5.77E-03
CFHR1	ENSG0000	5.77E-03
PROSER2-AS1	ENSG0000	5.78E-03
IL1RL1	ENSG0000	5.78E-03
RP11-594N15.3	ENSG0000	5.82E-03
BRINP1	ENSG0000	5.83E-03
CCNO	ENSG0000	5.83E-03
SYT4	ENSG0000	5.85E-03
LMOD3	ENSG0000	5.85E-03
AR	ENSG0000	5.85E-03
SCG2	ENSG0000	5.86E-03
PIM1	ENSG0000	5.87E-03
MAPK4	ENSG0000	5.89E-03
RAB12	ENSG0000	5.89E-03
TMEM200B	ENSG0000	5.89E-03
LCN6	ENSG0000	5.94E-03
VGLL3	ENSG0000	5.99E-03
AL022393.9	ENSG0000	5.99E-03
BRMS1L	ENSG0000	6.00E-03
RP11-368L12.1	ENSG0000	6.01E-03
FNDC1	ENSG0000	6.03E-03
RP11-433J20.1	ENSG0000	6.03E-03
DDIT4L	ENSG0000	6.03E-03

RP11-490O6.2	ENSG0000	6.05E-03
ZBTB16	ENSG0000	6.06E-03
THSD7A	ENSG0000	6.12E-03
LINC00856	ENSG0000	6.13E-03
TECTA	ENSG0000	6.14E-03
RP11-221N13.3	ENSG0000	6.19E-03
TAL1	ENSG0000	6.19E-03
C6	ENSG0000	6.24E-03
LINC00997	ENSG0000	6.26E-03
CTD-2033D15.2	ENSG0000	6.28E-03
LIPM	ENSG0000	6.29E-03
MYOZ3	ENSG0000	6.33E-03
ISLR2	ENSG0000	6.35E-03
GFRA3	ENSG0000	6.35E-03
VIPR2	ENSG0000	6.35E-03
RECK	ENSG0000	6.36E-03
RP11-286H14.8	ENSG0000	6.37E-03
SOCS3	ENSG0000	6.38E-03
ELANE	ENSG0000	6.38E-03
PITX2	ENSG0000	6.39E-03
AC124944.3	ENSG0000	6.39E-03
COLEC11	ENSG0000	6.44E-03
RET	ENSG0000	6.45E-03
RGS5	ENSG0000	6.45E-03
SNCA	ENSG0000	6.46E-03
IGSF21	ENSG0000	6.46E-03
DIRC3	ENSG0000	6.48E-03
RP11-136C24.3	ENSG0000	6.50E-03
