# Supplementary material for: Prognosis‐related gene signature is enriched in cancer‐associated fibroblasts in the stem‐like subtype of gastric cancer
Source: Clin Transl Med. 2022 Jun 26;12(6):e930. doi: 10.1002/ctm2.930 (PMC9234682; doi:10.1002/ctm2.930)
Supplement: Supplementary file 5 — Table S2. Activated fibroblast gene list [file CTM2-12-e930-s006.pdf]

ID
CILP
OGN
ELN
PTGS2
TIMP3
EGR2
COL14A1
SPON1
TNC
GABARAP
IGF1
TCEAL7
IGFBP5
MMP23B
COL3A1
THBS4
ASPN
COL8A1
COL6A3
MAFB
NEU1
CRABP2
TLE4
TNFAIP3
DIO2
MTRNR2L12
PABPC1
DPT
SFRP2
HES1
RPL36A
GADD45A
MEG3
MTRNR2L8
COL1A2
PABPC4
MRC2
TTC3
RPL17
TSHZ2
XIST
COL1A1

RPS26
RPS10
SPARC
SPON2
TCEAL9
CCDC80
MT-ND3
MXRA8
COL6A1
RPL39
NPM1
FOS
MT-ND5
EID1
FOSB
EEF1A1
RPS17
RPS4X
RPL26
RPL6
LUM
JUN
RPL5
ACTB
PTMA
MT-CO2
NEAT1
S100A4
CTGF
IGFBP7
C11orf96
MYL6
GSN
CALD1
CFD
HSPB1
IFITM3
GAPDH
CALM2
SERPING1
CEBPD
IFITM2
CXCL14

FILIP1L
CYR61
HLA-B
RARRES2
SOD3
FTH1
RHOB
TPM2
TPM1
RND3
PLA2G2A
HLA-A
MFGE8
B2M
LPP
C3
CXCL8
ID3
MYL9
SULF1
VMP1
BST2
MT1E
CRIP1
CXCL1
PHLDA2
HLA-C
TAGLN
CRYAB
MYLK
IFI27
TM4SF1
IGKC
ACTA2
MIF
MT1X
MT2A
MT1M
CD74
APOD
NDUFA4L2
PPP1R14A
G0S2

DEPP1
EPYC
IGHA1
ADIRF
MMP11
AL603756.1
IFITM1
MT1A
COL10A1
KRT8
GPX3
TFF3
S100P
PIGR
ACTG2
CCL19
MYH11
IGLC2
CST1
RGS5
COL11A1
PHGR1
KRT18
STC1
CXCL9
CST4
