# Supplementary material for: Prognosis‐related gene signature is enriched in cancer‐associated fibroblasts in the stem‐like subtype of gastric cancer
Source: Clin Transl Med. 2022 Jun 26;12(6):e930. doi: 10.1002/ctm2.930 (PMC9234682; doi:10.1002/ctm2.930)
Supplement: Supplementary file 6 — Supplementary material [file CTM2-12-e930-s001.docx]

**Supplementary material**

**Materials and Methods**

**Data and gene enrichment pathway analysis**

Bulk samples from four cohorts and single-cell gastric cancer data from two cohorts were used. First, 500 genes related to gastric cancer prognosis were selected using gepia2 (http://gepia2.cancer-pku.cn/#index) [1]. These top 500 genes were selected based on the *P* value. The accuracy of these SIG500 scores was increased by performing over 1,000,000 runs using the GSVA R package [2].

**Bulk sample analysis**

For the bulk sample, differential gene expression analysis was performed with high and low SIG500 samples using the limma R package [3]. To analyze the enriched pathways in the high SIG500 group, based on *P* value = 0.05 as the input, gene values were analyzed using METASCAPE [4]. For survival analysis, R package “survival” and “survminer” were employed. Furthermore, CIBERSORT [5] was used to analyze the immune landscape, and 50 cancer hallmark [6] gene sets were downloaded from MSigDB (http://www.gsea-msigdb.org/gsea/msigdb/). For each signature score, the enrichment of a single sample was calculated using the GSVA R package [2]. The molecular subtype of gastric cancer is classified according to the following characteristics: 1) immune subtype is defined as positive expression of both GZMB (granzyme B) and WARS (tryptophan tRNA synthetase); 2) epithelial subtype is defined as positive expression of CDX1 (caudal type homeobox 1); 3) stem-like subtype is defined as positive expression of SFRP4 (secreted frizzled related protein). Clinically, immune subtype harbors the most favorable prognosis and is not responsive to chemotherapy. Epithelial subtype has intermediate prognosis and is the subtype sensitive to chemotherapy. Stem-like subtype has the worst prognosis among other subtypes and is generally not responsive to standard chemotherapy or immune-directed therapy [7].

Additionally, the expression profiling of the patients with gastric cancer at the Samsung Medical Center was downloaded from <http://tide.dfci.harvard.edu/login/> to analyze the results of immunotherapy response according to SIG500 status in gastric cancer [8].

**Single-cell analysis**

Two single-cell cohorts were used. First, data from one cohort obtained from the Ji Research Group was downloaded from https://dna-discovery.stanford.edu/research/datasets/ [9], and quality control (QC) was performed according to our strict standards instead of the published QC methods [9]. Cells that satisfied the following criteria were filtered out for downstream analysis: (1) cells with unique feature counts of < 200 or > 2500 (in the outlier range, because the latter could indicate probable doublets); (2) cells with mitochondrial counts of more than 5%. (Seurat default). Thereafter, the retained cells were classified into eight types using a published marker gene [9]. For the second cohort comprising single-cell data, tumor samples from published studies were used [10]. Strict QC was not performed separately; only tumor samples were used, and the marker gene was selected following published standards. The Seurat R package [11] was used for single-cell analysis, and RaceID and StemID [12] were used to calculate the stemness of each cell type. All statistical tests were performed in R (version 4.1.2).

**Signal transduction pathway and drug–target analysis**

NicheNet [13] was used to analyze ligand-receptor modeling and intercellular communication by linking ligands to the target gene set at the single-cell level. In addition, drug–target interaction was analyzed using ConsensusPathDB-human (CPDB) [13], and drug reactivity was evaluated based on Genomics of Drug Sensitivity in Cancer (GDSC) [14].

1. Tang, Z., et al., *GEPIA2: an enhanced web server for large-scale expression profiling and interactive analysis.* Nucleic Acids Res, 2019. **47**(W1): p. W556-W560.

2. Hanzelmann, S., R. Castelo, and J. Guinney, *GSVA: gene set variation analysis for microarray and RNA-seq data.* BMC Bioinformatics, 2013. **14**: p. 7.

3. Ritchie, M.E., et al., *limma powers differential expression analyses for RNA-sequencing and microarray studies.* Nucleic Acids Res, 2015. **43**(7): p. e47.

4. Zhou, Y., et al., *Metascape provides a biologist-oriented resource for the analysis of systems-level datasets.* Nat Commun, 2019. **10**(1): p. 1523.

5. Newman, A.M., et al., *Determining cell type abundance and expression from bulk tissues with digital cytometry.* Nat Biotechnol, 2019. **37**(7): p. 773-782.

6. Hanahan, D. and R.A. Weinberg, *Hallmarks of cancer: the next generation.* Cell, 2011. **144**(5): p. 646-74.

7. Cheong, J.H., et al., *Predictive test for chemotherapy response in resectable gastric cancer: a multi-cohort, retrospective analysis.* Lancet Oncol, 2018. **19**(5): p. 629-638.

8. Kim, S.T., et al., *Comprehensive molecular characterization of clinical responses to PD-1 inhibition in metastatic gastric cancer.* Nat Med, 2018. **24**(9): p. 1449-1458.

9. Sathe, A., et al., *Single-Cell Genomic Characterization Reveals the Cellular Reprogramming of the Gastric Tumor Microenvironment.* Clin Cancer Res, 2020. **26**(11): p. 2640-2653.

10. Kim, J., et al., *Single-cell analysis of gastric pre-cancerous and cancer lesions reveals cell lineage diversity and intratumoral heterogeneity.* NPJ Precis Oncol, 2022. **6**(1): p. 9.

11. Hao, Y., et al., *Integrated analysis of multimodal single-cell data.* Cell, 2021. **184**(13): p. 3573-3587 e29.

12. Grun, D., et al., *De Novo Prediction of Stem Cell Identity using Single-Cell Transcriptome Data.* Cell Stem Cell, 2016. **19**(2): p. 266-277.

13. Browaeys, R., W. Saelens, and Y. Saeys, *NicheNet: modeling intercellular communication by linking ligands to target genes.* Nat Methods, 2020. **17**(2): p. 159-162.

14. Qin, Y., et al., *A tool for discovering drug sensitivity and gene expression associations in cancer cells.* PLoS One, 2017. **12**(4): p. e0176763.
